# Supplementary material for: MolDiscovery: learning mass spectrometry fragmentation of small molecules
Source: Nat Commun. 2021 Jun 17;12:3718. doi: 10.1038/s41467-021-23986-0 (PMC8211649; doi:10.1038/s41467-021-23986-0)
Supplement: Supplementary file 1 — Supplementary Information [file 41467_2021_23986_MOESM1_ESM.pdf]

Supplementary Information

MolDiscovery: Learning Mass Spectrometry Fragmentation of Small Molecules

Liu Cao[1,†], Mustafa Guler[1,†], Azat Tagirdzhanov[2,3], Yiyuan Lee[1], Alexey Gurevich[2], Hosein Mohimani[1,*]

[†]These authors contributed equally: Liu Cao, Mustafa Guler
[1]Carnegie Mellon University, Pittsburgh, 15213, United States of America
[2]St. Petersburg State University, St. Petersburg, 199004, Russia
[3]St. Petersburg Electrotechnical University "LETI", St. Petersburg, 197376, Russia
[*]Corresponding Author. Email: hoseinm@andrew.cmu.edu

1 Supplementary Notes

Supplementary Note 1. Parameter settings for benchmarking.

Running CFM-ID. We used CFM-ID 2.0 associated with R31 at https://sourceforge.net/p/cfm-id/code/HEAD/tree/. Preprocessing consisted of converting compounds to predicted spectra using the cfm-predict binary. Following directions on the CFM-ID wiki (https://sourceforge.net/p/cfm-id/wiki/Home/#cfm-predict) we ran

```
cfm-predict SMILES 0.001 param_output0.log param_config.txt OUT_DIR 1 1
```

All parameters here are the defaults except that the supress_exceptions option was turned on. As suggested by the FAQ (https://sourceforge.net/p/cfm-id/wiki/Home/#frequently-asked-questions) we used the metab_se_cfm parameters. Scoring was done with the cfm-id-precomputed binary. According to the same FAQ, we repeated our single-energy data for each of the energy levels. We outputted 10 candidates using a relative tolerance of 10ppm and an absolute tolerance of 0.01 Da using the Jaccard (suggested for ESI-MS/MS) scoring method. Since CFM-ID does not have in-built filtering of a chemical database by precursor m/z we have added our own filtering to create a custom database for each spectrum, considering all compounds with mass within 0.02 Da of the mass of a [M+H]+ adduct for the current spectrum.

Running MAGMa+. We used MAGMa+ 1.0.1. To preprocess our chemical compound database we created an SQLite database containing a single table using the following SQL command:

```
CREATE TABLE molecules ( id TEXT PRIMARY KEY, mim INTEGER NOT NULL,
charge INTEGER NOT NULL, natoms INTEGER NOT NULL, molblock TEXT,
inchikey TEXT, molform TEXT, name TEXT, reference TEXT, logp INT );
```

To fully replicate MAGMa+ we then built the same index they do after inserting all compounds using the SQL command:

```
PRAGMA temp_store = 2;
CREATE INDEX idx_cover ON molecules ( charge, mim, natoms, reference,
molform, inchikey, name, molblock, logp );
```

MAGMa+ does not support the MGF format. Each MGF spectrum was converted to MAGMa+ internal mass tree format via the following process: (i) peak intensities were normalized to range from 0 to 100, (ii) the precursor m/z was set to intensity 100, and (iii) mass tree was constructed using format 'PRECURSOR: 100 (mz1: norm_intensity1, mz2: norm_intensity2, ...)'

Each individual spectrum in the mass tree format was read via the read_ms_data subcommand using positive ionization mode and a precursor absolute error tolerance of 0.02 Da

and a maximum product ion error tolerance of 0.01 Da. Each spectrum was then annotated with the annotate subcommand using the parameter minimum peak intensity set to 0.

Running CSI:FingerID. We used SIRIUS 4.5.3 with SIRIUS lib 4.4.8 and CSI:FingerID 1.4.8. Compounds were preprocessed into a custom database using the custom-db option with a file with one SMILES per line. Options were set by exporting a command-line version of an analysis set using the SIRIUS GUI, command replicated below with placeholders for inputs.

```
sirius \
    -i $SINGLE_MGF_SPECTRUM \
    -o $OUTPUT_DIR \
    config \
    --AlgorithmProfile qtof \
    --IsotopeMs2Settings IGNORE \
    --MS2MassDeviation.allowedMassDeviation "10.0ppm (0.01 Da)" \
    -NumberOfCandidatesPerIon 1 \
    --Timeout.secondsPerTree 1000 \
    --NumberOfCandidates 10 \
    --FormulaSettings.enforced HCNOPS \
    --Timeout.secondsPerInstance 0\
    --AdductSettings.detectable "[[M+H]+]" \
    --StructureSearchDB $STRUCTURE_DB_NAME \
    --AdductSettings.fallback "[[M+H]+]" \
    --FormulaResultThreshold true \
    --RecomputeResults true \
    formula \
    structure
```

Running MetFrag. We used version 2.4.5 of the MetFragCommandLine jar from the MetFragRelaunched repository. We used a slightly modified version of the parameter file used for the MetFrag submission in CASMI 2016, reproduced below.

```
#
# database parameters -> how to retrieve candidates
#
#
MetFragDatabaseType = LocalPSV
LocalDatabasePath = $PSV_FILE
#
#
peak matching parameters
#
```

```
FragmentPeakMatchAbsoluteMassDeviation = 0.01
FragmentPeakMatchRelativeMassDeviation = 5
PrecursorIonMode = 1
IsPositiveIonMode = True
#
# scoring parameters
#
# taken from CASMI 2016 positive mode
NumberMaximumPeaksUsed = 40
FingerprintPeakAnnotationFile = $METFRAG_BASE/peak_annotations_pos.txt
FingerprintLossAnnotationFile = $METFRAG_BASE/loss_annotations_pos.txt
MetFragScoreTypes = FragmenterScore,
AutomatedPeakFingerprintAnnotationScore,
AutomatedLossFingerprintAnnotationScore
MetFragScoreWeights = 0.378258289605048,
0.487761785135587,
0.133979925259365

FingerprintType = CircularFingerprinter
LossFingerprintAnnotationBetaValue = 5e-04
LossFingerprintAnnotationAlphaValue = 0.0025
PeakFingerprintAnnotationBetaValue = 0.0125
PeakFingerprintAnnotationAlphaValue = 1e-04
#
# output
# SDF, XLS, CSV, ExtendedXLS, ExtendedFragmentsXLS
#
MetFragCandidateWriter = CSV
#
# following parameteres can be kept as they are
#
MaximumTreeDepth = 2
MetFragPreProcessingCandidateFilter =
UnconnectedCompoundFilter,IsotopeFilter
MetFragPostProcessingCandidateFilter = InChIKeyFilter
NumberThreads = 1
# Adding for each spectrum:
# PeakListPath
# IonizedPrecursorMass
# DatabaseSearchRelativeMassDeviation
# SampleName
# ResultsPath
```

Supplementary Note 2. Fragmentation graph construction algorithm.

Algorithm 1 Fragmentation Graph Construction Algorithm

Input: Metabolite graph $metGraph$, maximum depth $maxDepth$
Output: Fragmentation graph FG

$root \leftarrow [1, \ldots, 1]$ // root contains all nodes in $metGraph$
$depth_1Frags \leftarrow \{frag \mid frag \in \text{HopcroftTarjan}(metGraph)\}$
$FGNodes \leftarrow \{root\} \cup depth_1Frags$
$FGEdges \leftarrow \{(root, frag) \mid frag \in depth_1Frags\}$
$prevDepthFrags \leftarrow depth_1Frags$
for $i \in \{2, \ldots, maxDepth\}$ **do**
  $currDepthFrags \leftarrow \emptyset$
  for all $parentFrag \in prevDepthFrags$ **do**
    for all $depth_1Frag \in depth_1Frags$ **do**
      $newFrag \leftarrow parentFrag \,\&\, depth_1Frag$
      $currDepthFrags \leftarrow currDepthFrags \cup \{newFrag\}$
      $FGNodes \leftarrow FGNodes \cup \{newFrag\}$
      $FGEdges \leftarrow FEdges \cup \{(parentFrag, newFrag)\}$
    end for
  end for
  $prevDepthFrags \leftarrow currDepthFrags$
end for
return $(FGNodes, FGEdges)$

Supplementary Note 3. LogRank computation.

Equation (1) shows the formula used to compute the *logRank* of a peak from its *rank*. Note that multiple peaks with different ranks can be assigned to the same *logRank*. Additionally there are only 8 possible values of *logRank* ($logRank = 0$ is reserved for the root node). For example, $logRank = 1$ represents the most intense peak, $logRank = 2$ represents the next 2 most intense peaks, and $logRank = 3$ represents the next 4 most intense peaks. In general, the peaks with rank between 2^{i-1} to $2^i - 1$ will be assigned to $logrank = i$. We define the lowest (farthest from rank 1) possible *logRank* as 7 and all peaks that would be assigned to a *logRank* larger than 7 are instead assigned to $logRank = 7$. We use this strategy to reduce the number of parameters in our model, and avoid overfitting. Note that using *logRank*, the number of parameters of the model reduces from 64 per *bondType* to only 7 per *bondType*.

$$logRank = \min(\lfloor \log_2(rank) \rfloor + 1, 7) \tag{1}$$

Fluctuations in peak intensity due to noise could change the annotated *logRank*. However, *logRank*s are more robust to these fluctuations in compare to ranks as the slight change of peak intensity will leads to a small change (at most 1) in *logRank*. In addition, the change of probability score due to the change of *logRank* is smooth (Supplmentary Fig. 1).

Supplementary Note 4. Running molDiscovery on GNPS.

To run molDiscovery on GNPS, please visit `https://gnps.ucsd.edu/ProteoSAFe/index.jsp?params=%7B%22workflow%22:%22MOLDISCOVERY%22%7D` (see below). To run molDiscovery in the command line, please visit `https://github.com/mohimanilab/molDiscovery` for details.

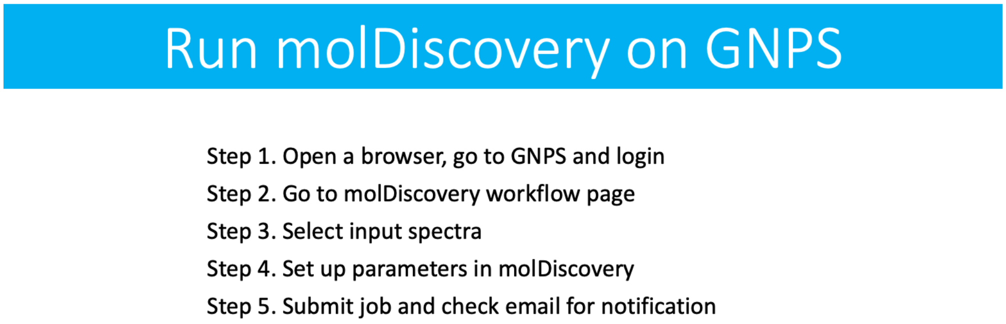

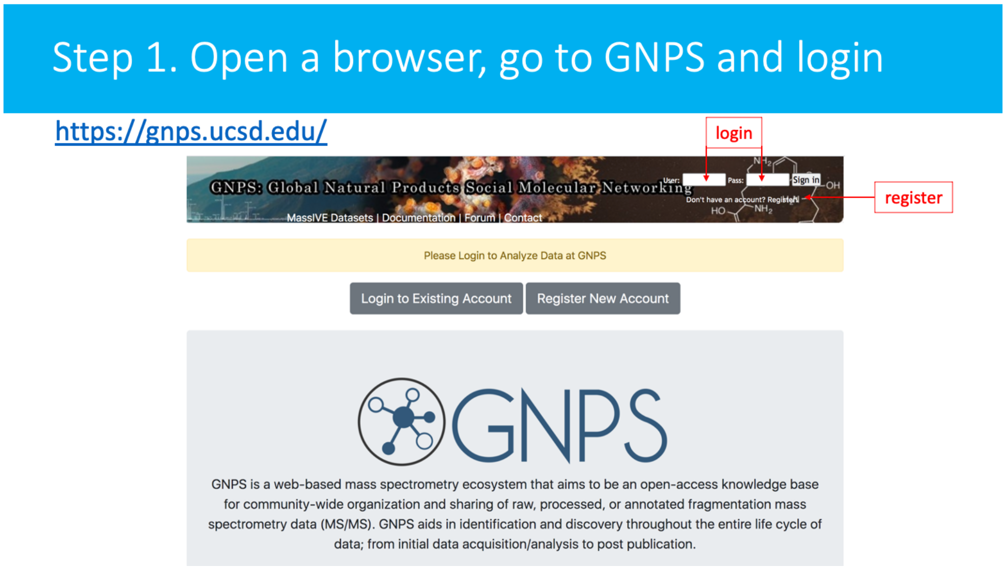

Step 2. Go to molDiscovery workflow page

https://gnps.ucsd.edu/ProteoSAFe/index.jsp?params=%7B%22workflow%22:%22MOLDISCOVERY%22%7D

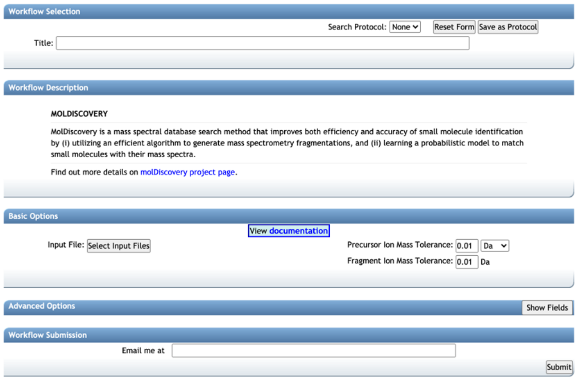

Step 3. Select input spectra

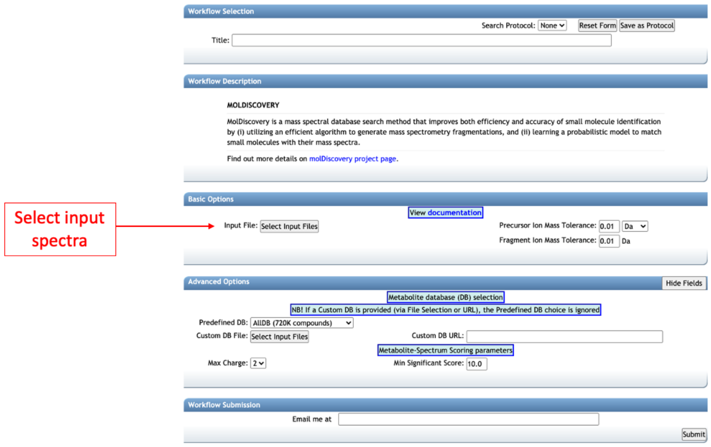

Step 3.1 Add input spectra to user directory

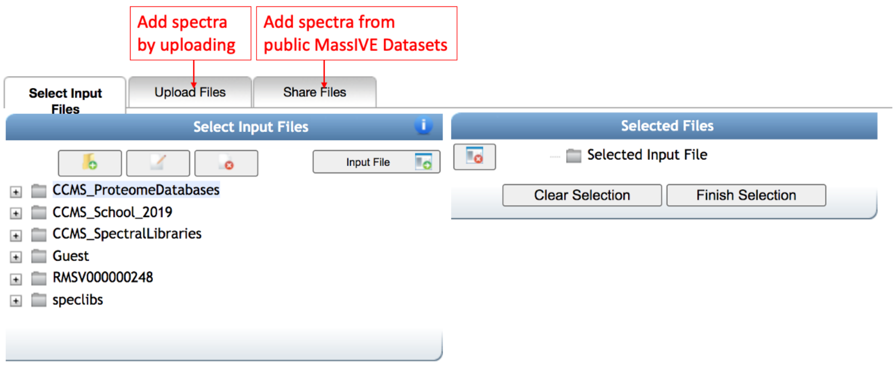

Step 3.2 Select input spectra from user directory

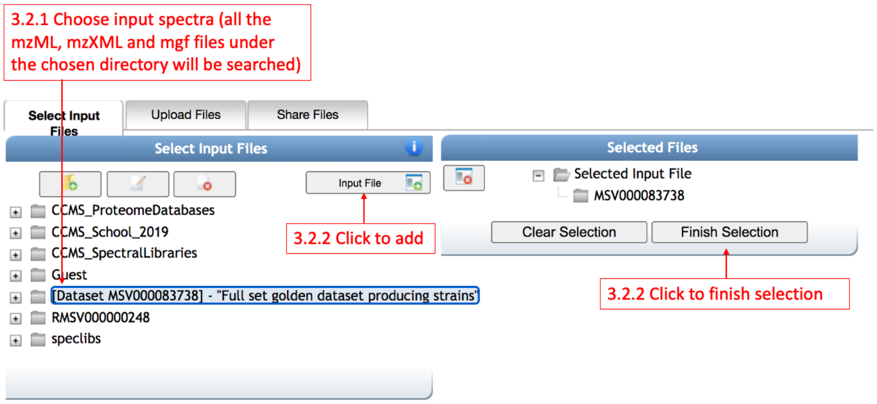

Step 4. Set up parameters in molDiscovery

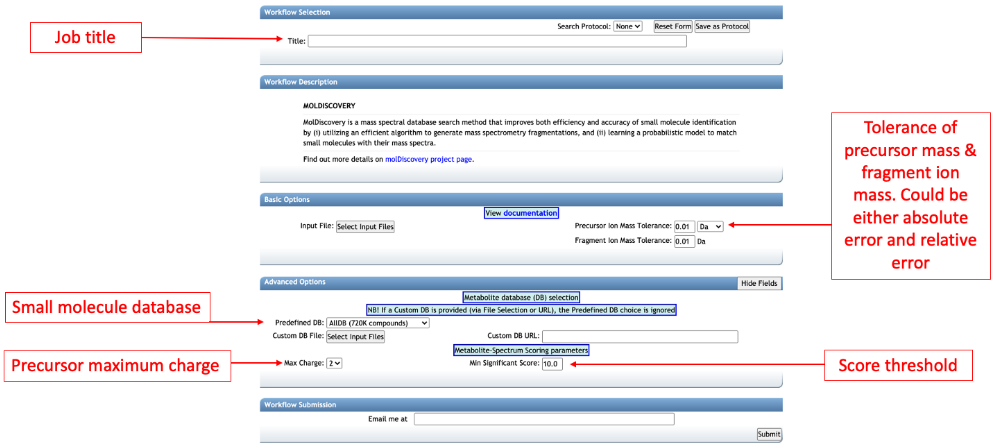

Step 5. Submit job and check email for notification

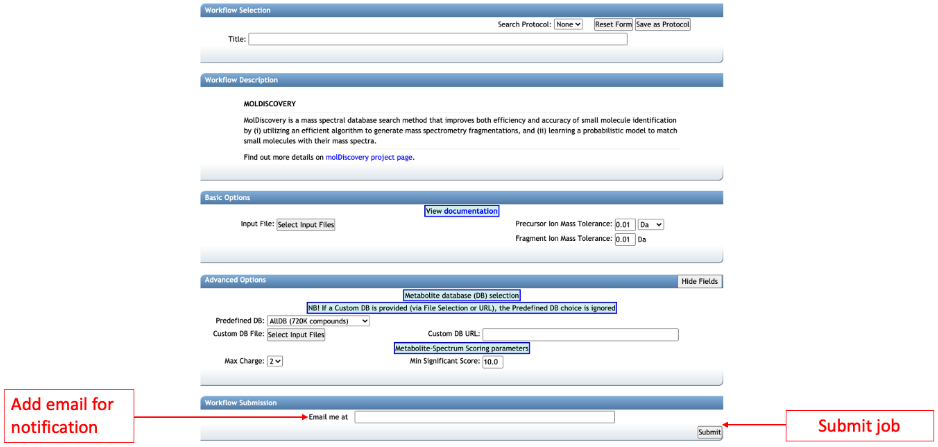

2 Supplementary Figures

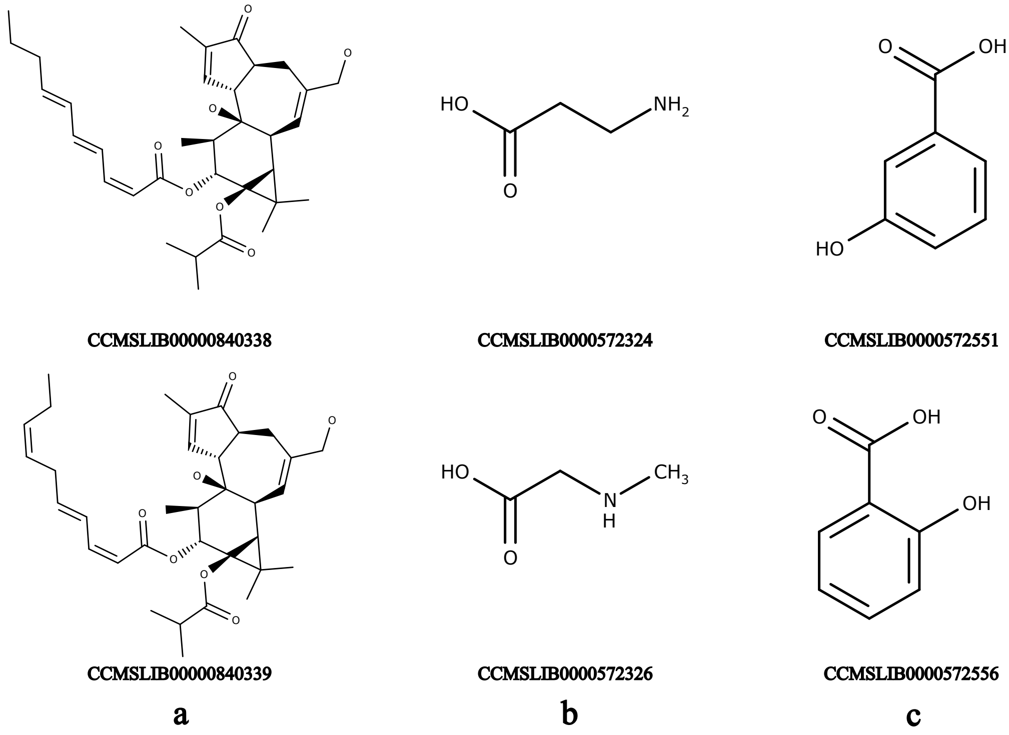

Supplementary Fig 1: Examples of different structures corresponding to the identical spectra in the GNPS spectral library. **a** splash10-00ei-0119850000-7e4cfe9a3a48492095dd, **b** splash10-0ab9-9000000000-41fe0c437d174e817929, and **c** splash10-0udi-9000000000-dc3aaeb701482eda1fe7.

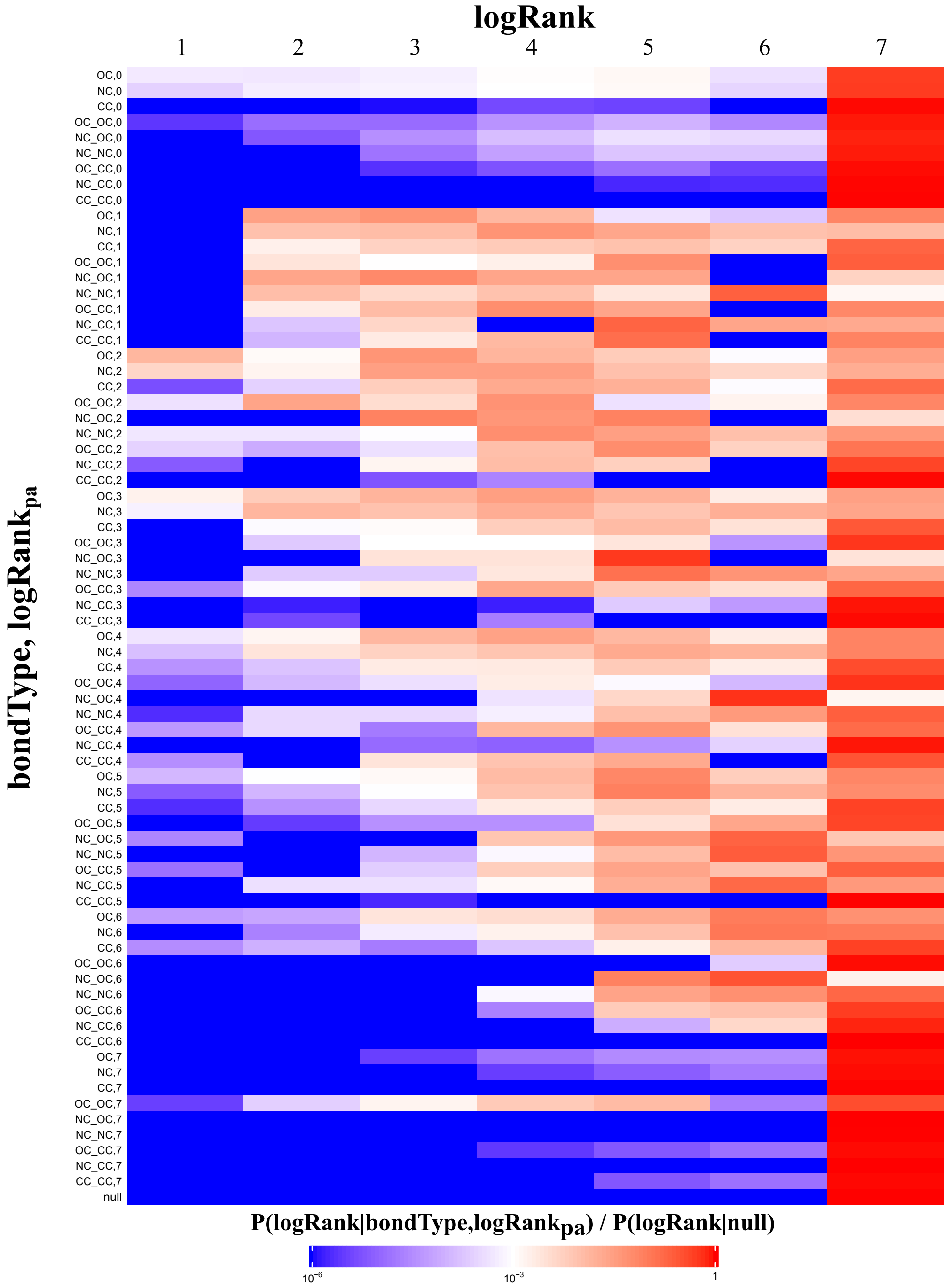

Supplementary Fig 2: Heatmap of $P(logRank|bondType, logRank_{pa})$ for charge +1 fragments. Each row represents $bondType$ and $logRank_{pa}$. The row "null" refers to the null distribution $P(logRank|null)$. Each column represents the $logRank$ of a child fragment. When $logRank_{pa}$ is 0, it means the parent is the root (precursor molecule).

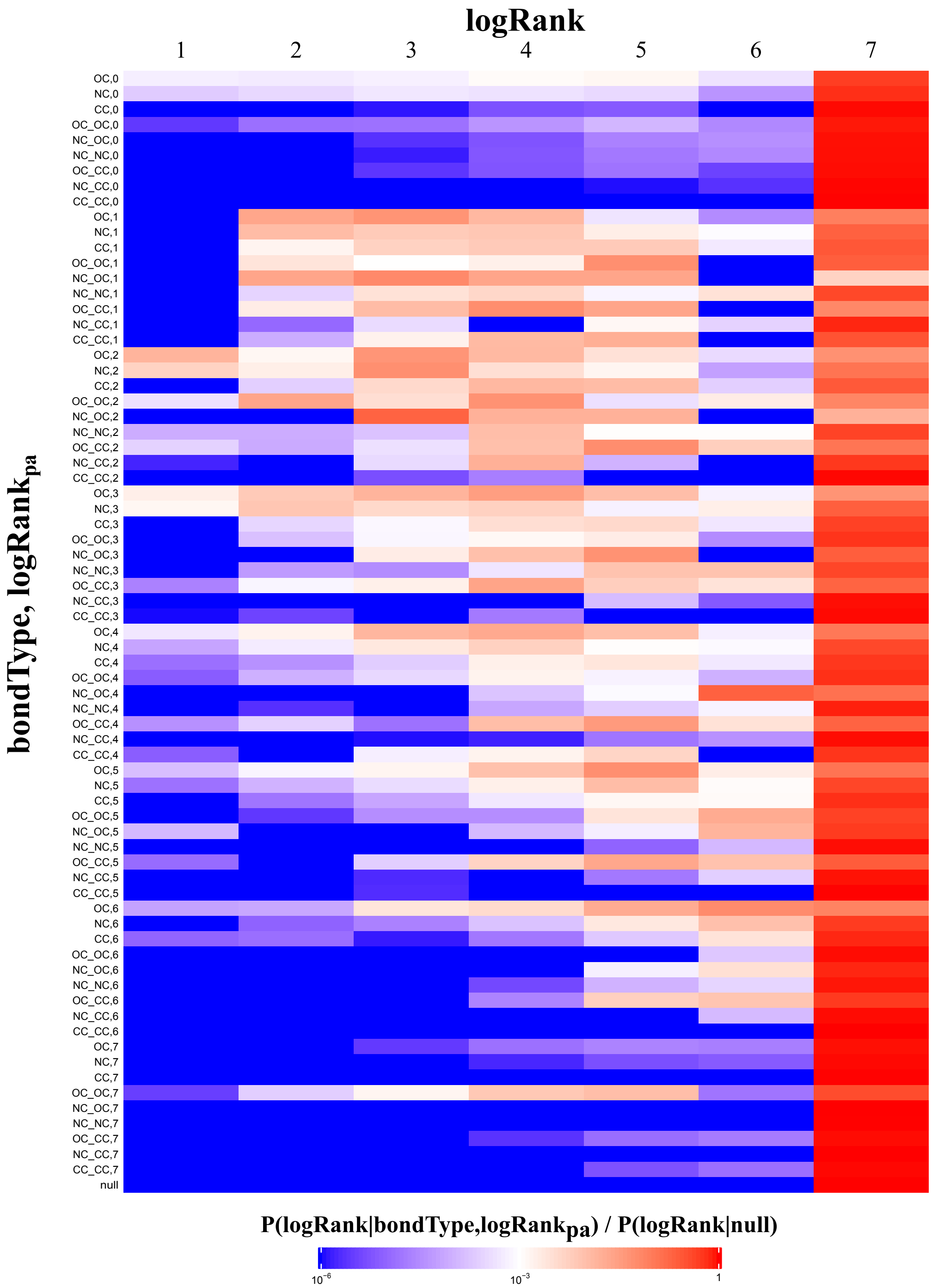

Supplementary Fig 3: Heatmap of $P(logRank|bondType, logRank_{pa})$ for charge +2 fragments. Each row represents $bondType$ and $logRank_{pa}$. The row "null" refers to the null distribution $P(logRank|null)$. Each column represents the $logRank$ of a child fragment. When $logRank_{pa}$ is 0, it means the parent is the root (precursor molecule).

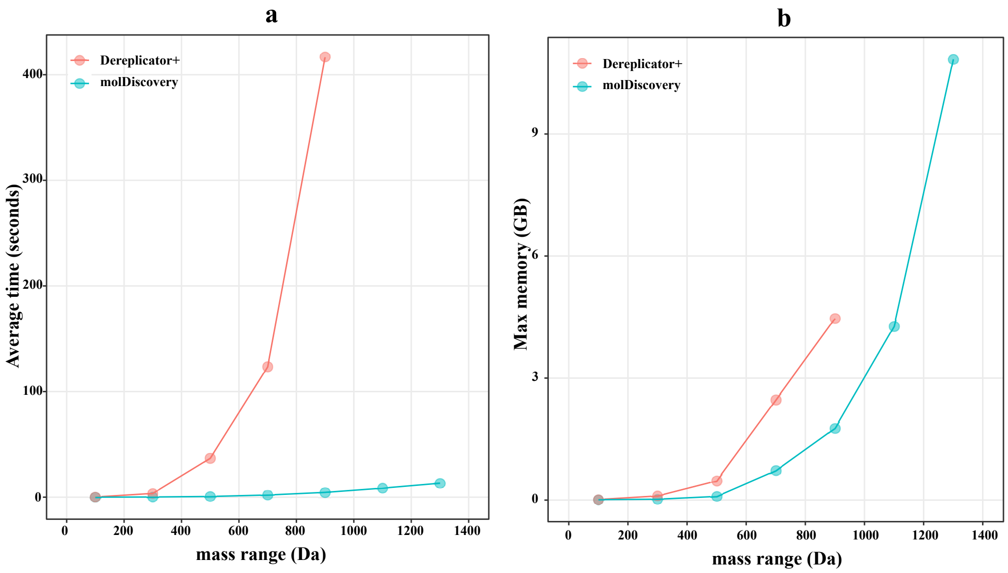

Supplementary Fig 4: Benchmarking fragmentation tree construction algorithms. We compare the **a** average fragmentation tree construction time and **b** maximum memory (resident set size) consumption of molDiscovery with Dereplicator+ on the DNP database over molecules of various masses. Note that we randomly selected 200 molecules from the DNP database for each 200 Da mass window to compute average running time per molecule as well as maximum memory usage. For cases exceeding the memory limit of 15 GB, no data point is shown.

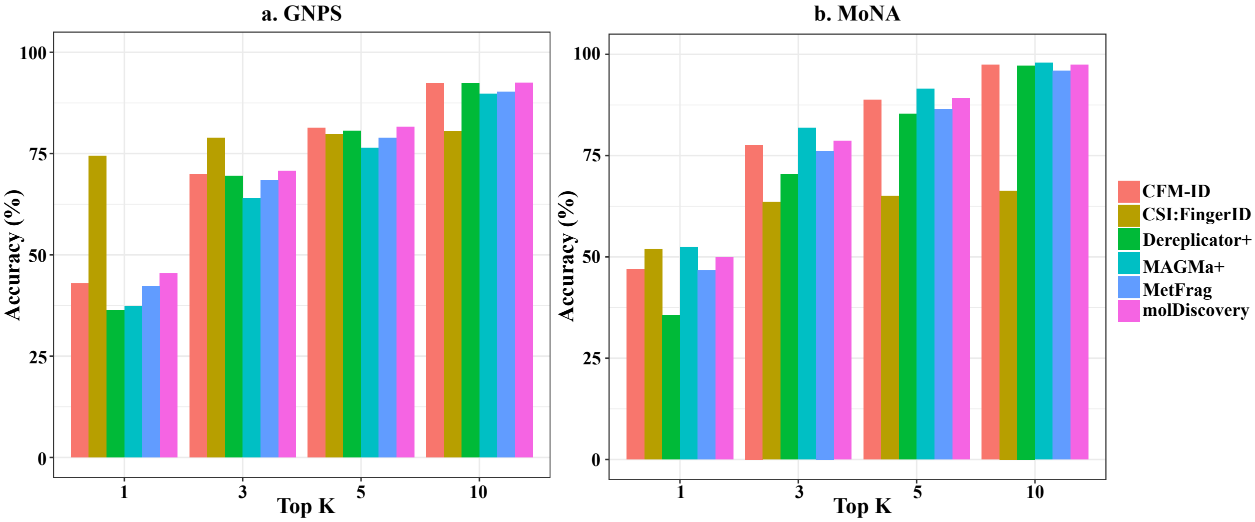

Supplementary Fig 5: Top 1, 3, 5, and 10 accuracy for all tested methods when searching **a** 4,437 spectra from the GNPS spectral library against 77,057 molecules from DNP and **b** 6,528 spectra from MoNA against 10,124 compounds from MoNA. Ties in scores were evaluated by setting the rank of the candidate compounds with tied scores to the average of all of those ranks. Note that 95.6% of GNPS spectra and 94.8% of MoNA spectra came from compounds found in the CSI:FingerID training dataset.

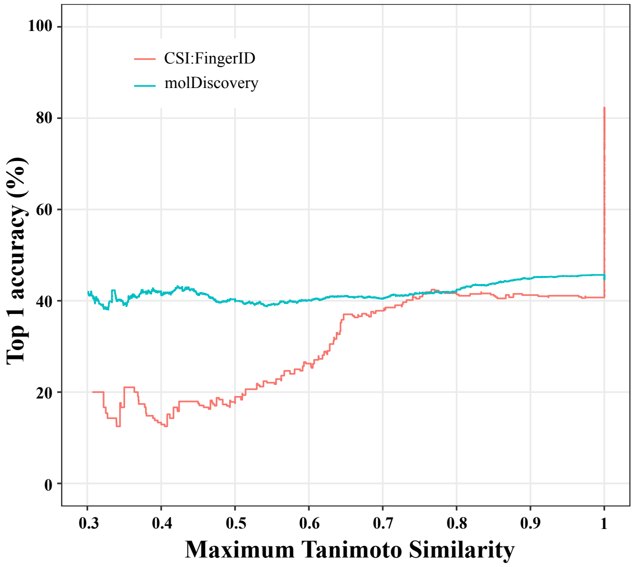

Supplementary Fig 6: Database search accuracy according to the Tanimoto similarity between training compounds and testing compounds. Note that there is a large jump in accuracy for CSI:FingerID when we reach a Tanimoto similarity of 1.0, indicating that CSI:FingerID is biased towards the molecular structures similar to its training data.

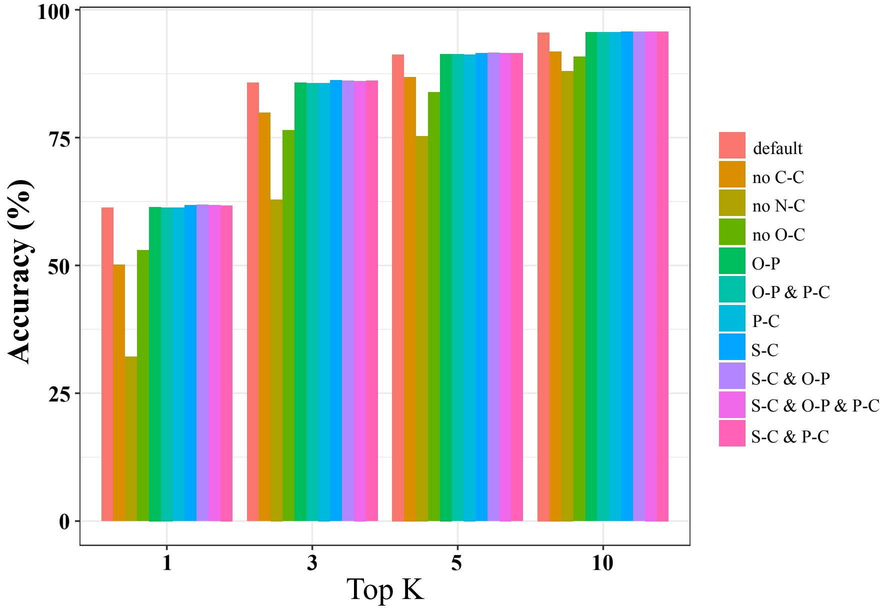

Supplementary Fig 7: Top K ($K = 1, 3, 5, 10$) accuracy of molDiscovery on a subset of high-resolution NIST20 spectra from QTOF instrument types. The default setting denotes the C-C, N-C, and O-C bond types. The effect of removing bond types (no O-C, no N-C, no C-C) and adding new bond types (S-C & P-C, S-C & O-P & P-C, S-C & O-P, S-C, P-C, O-P & P-C, O-P) are explored. Removing any of our default bond types results in a severe drop in top 1 accuracy (8.3% for C-C, 29.2% for N-C). Addition of P-C, S-C and O-P bonds does not improve the overall accuracy.

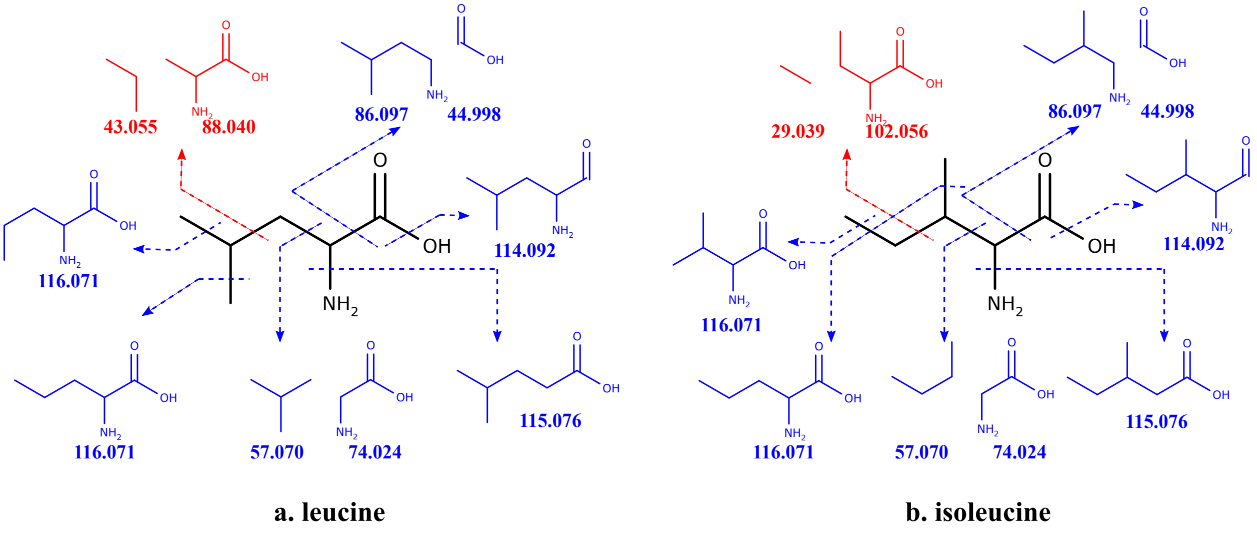

Supplementary Fig 8: Depth 1 fragmentations of **a** leucine and **b** isoleucine by molDiscovery. The shared masses of the fragments are in blue, while the different masses are in red.

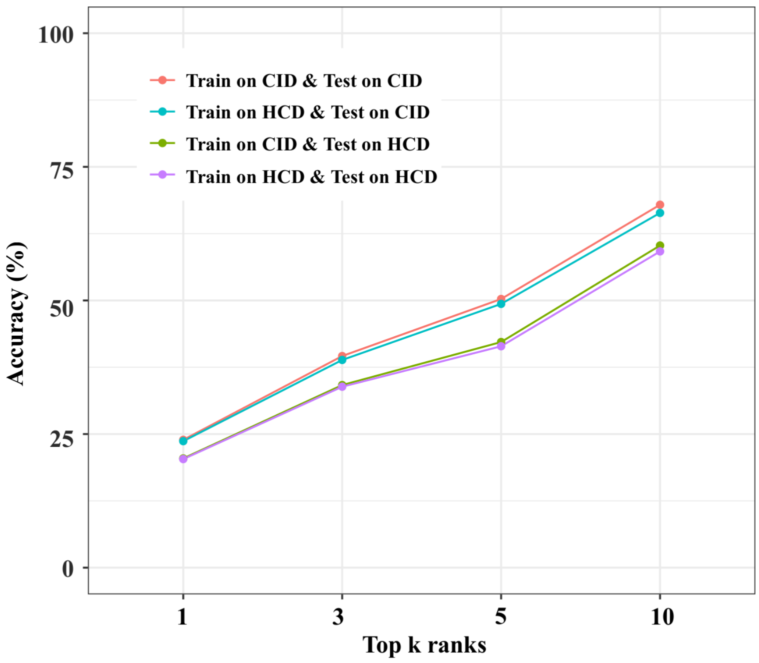

Supplementary Fig 9: Accuracy of molDiscovery on tandem mass spectra of different fragmentation modes. MolDiscovery models were trained on the GNPS spectra augmented with MoNA spectra labeled with either CID or HCD fragmentation modes. The two models are then tested on both CID and HCD data. Matches were ranked according to score. The fraction of spectra for which the correct molecule was in the top k ($k = 1, 3, 5, 10$) matches are shown along the y-axis.

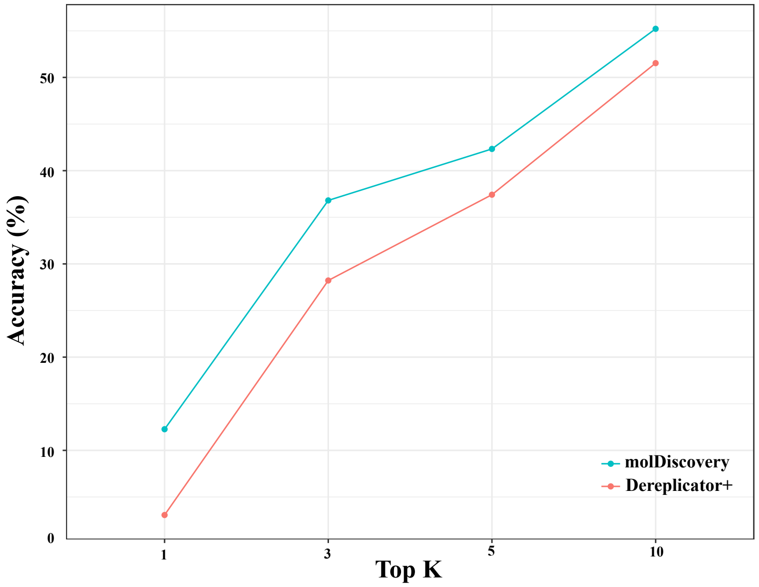

Supplementary Fig 10: The top $K = 1, 3, 5, 10$ database search accuracies of Dereplicator+ and molDiscovery on 163 doubly-charged spectra from MoNA. The molDiscovery model was initially trained on 4,781 singly-charged spectra from GNPS and further trained on 813 spectra identified as doubly-charged spectra by Dereplicator+.

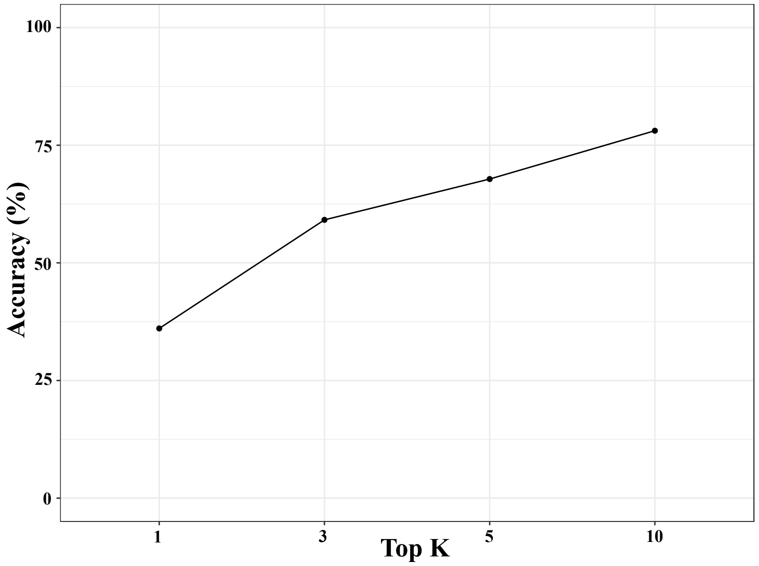

Supplementary Fig 11: Database search accuracies of molDiscovery on 3964 spectra of negative ionization mode against the unique DNP database. Here, the precursor mass tolerance is 0.02 and the product ion mass tolerance is 0.01. The top $K = 1, 3, 5, 10$ accuracies are 36%, 59%, 68% and 78%, respectively. Note that we did not retrain molDiscovery on negatively charged spectra.

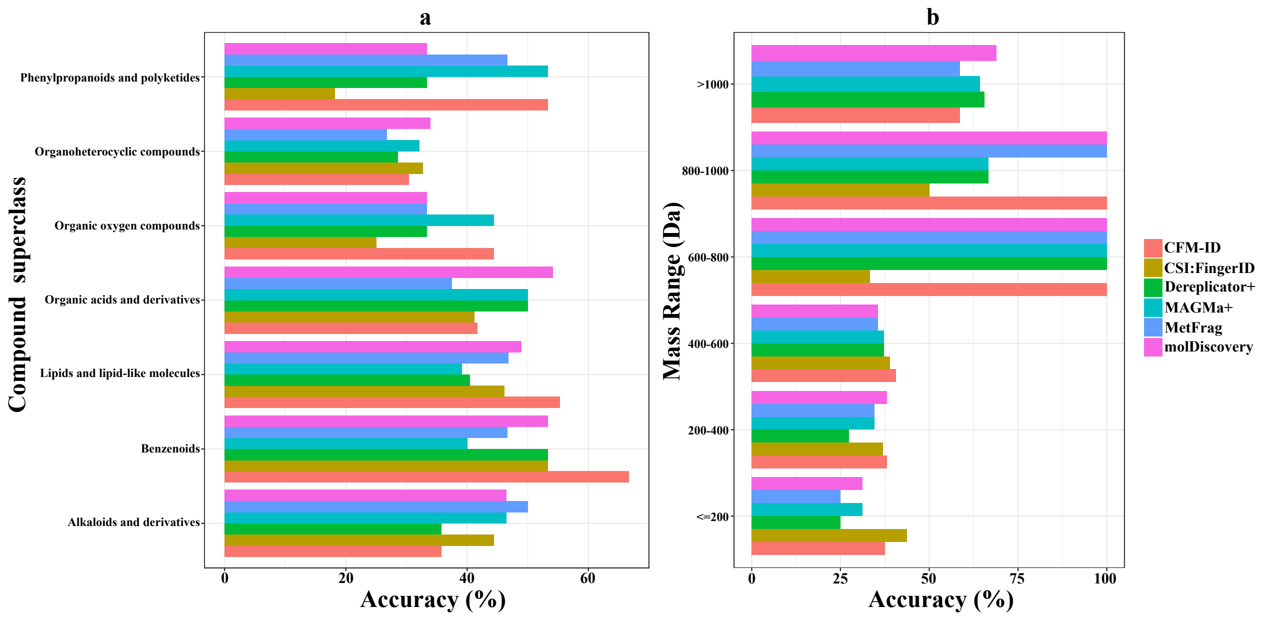

Supplementary Fig 12: Database search accuracy of molDiscovery, Dereplicator+, CSI:FingerID, MAGMa+, and CFM-ID on molecules of **a** different superclasses from ClassyFire and **b** different mass ranges on the GNPS spectral library compounds excluding CSI:FingerID training data. See Supplementary Fig. 13 for the number of compounds in each class and mass range.

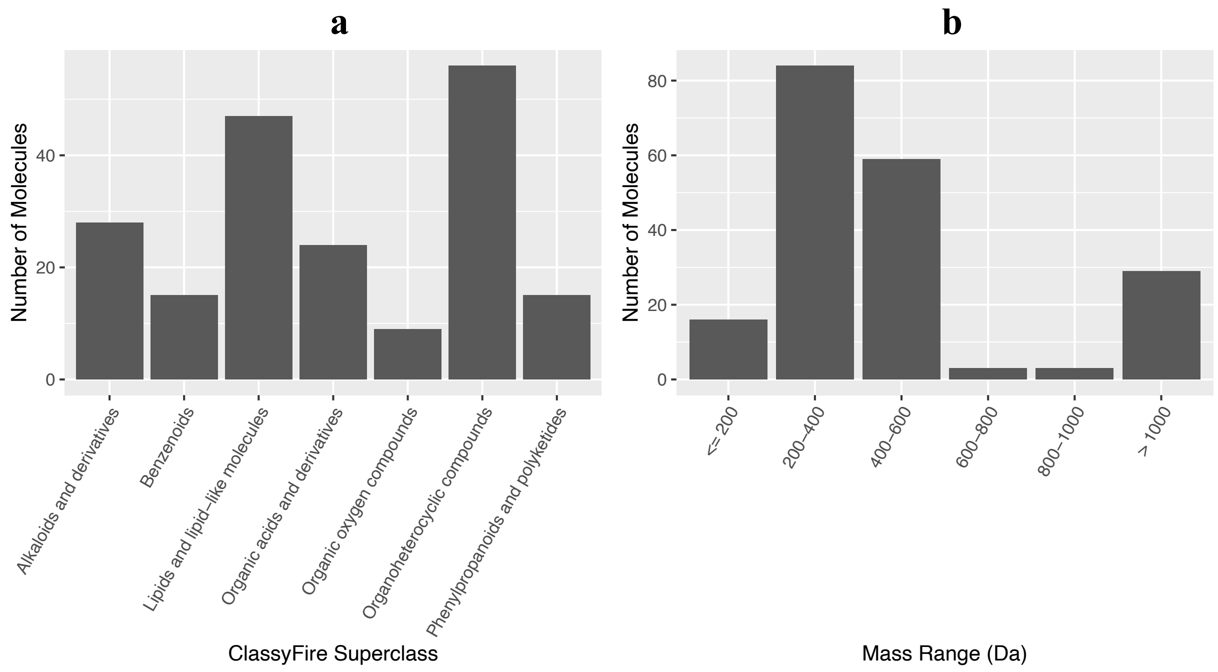

Supplementary Fig 13: Number of compounds in each division of the non-redundant GNPS dataset. **a** Number of compounds classified as each superclass found by ClassyFire. **b** Number of compounds that fall within different mass ranges.

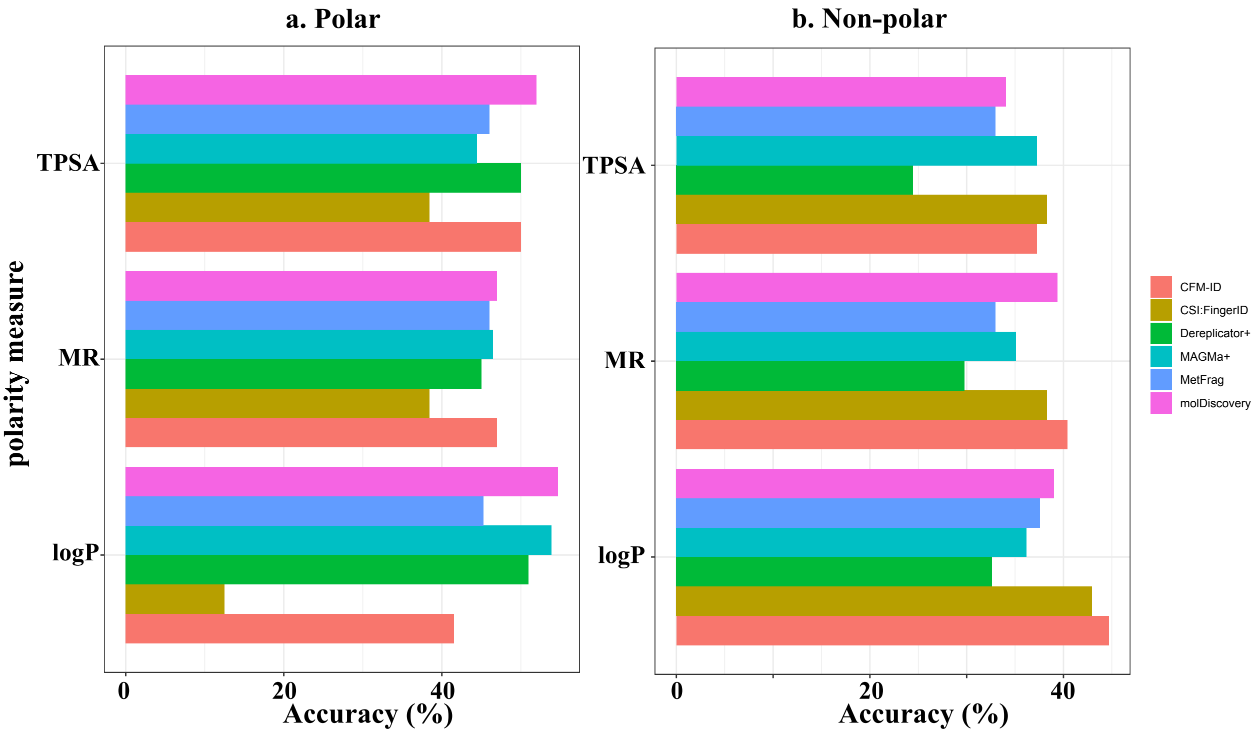

Supplementary Fig 14: Database search accuracy of **a** polar and **b** non-polar compounds. Three polarity measures predicted by the RDKit package, including topological polar surface area (TPSA), molecular refractivity (MR), and partition coefficient (logP), are used to classify a compound as polar and non-polar. Here, if MR or TPSA is greater than median value of all the testing compounds, or logP is less than 1, then the compound is called polar.

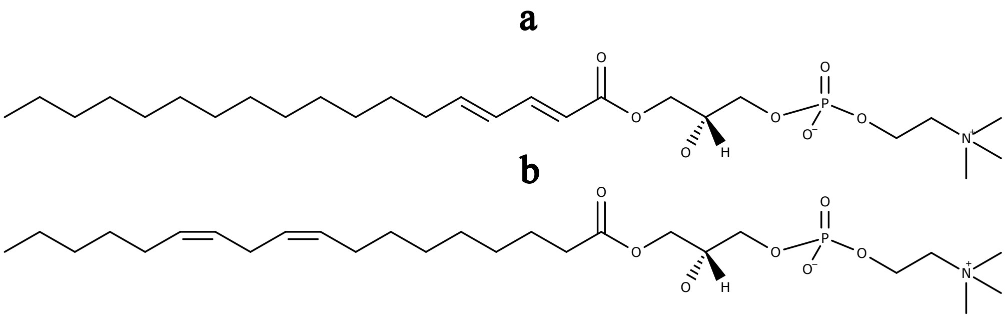

Supplementary Fig 15: Structure of very similar lipids **a** LMGP01050034 and **b** LMGP01050035. Both compounds are candidates for spectrum CCMSLIB00003087761 from the PNNL lipids library. The correct compound **a** gets a score of -9.63 while the incorrect compound **b** gets a score of -7.42. Many of the lipid structures are very similar to each other, making it more difficult to distinguish them based on mass spectra

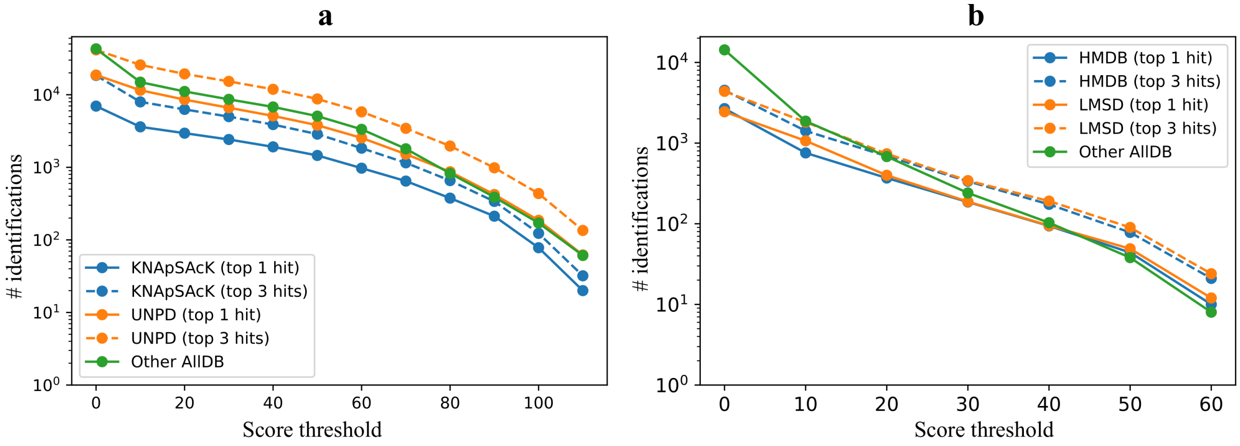

Supplementary Fig 16: MolDiscovery results on **a** plant and **b** human serum datasets. The number of molecule-spectrum matches at various score thresholds in the searches against **a** KNApSAcK, UNPD and the rest of AllDB, and **b** HMDB, LMSD and the rest of AllDB. For KNApSAcK, UNPD , HMDB, and LMSD both top 1 (solid lines) and top 3 (dashed lines) accuracy identifications are counted.

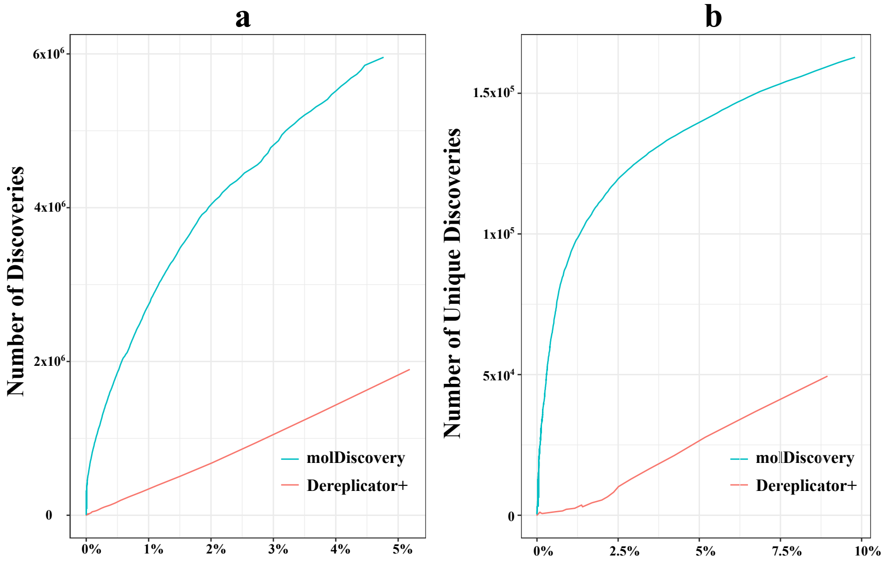

Supplementary Fig 17: Performance of molDiscovery and Dereplicator+ on GNPS spectral datasets. The curves show the number of **a** small molecule-spectrum matches and **b** unique compounds identified by molDiscovery and Dereplicator+ in the search of 8 million spectra from 46 GNPS spectral datasets against 719,958 compounds of AllDB at different FDR levels.

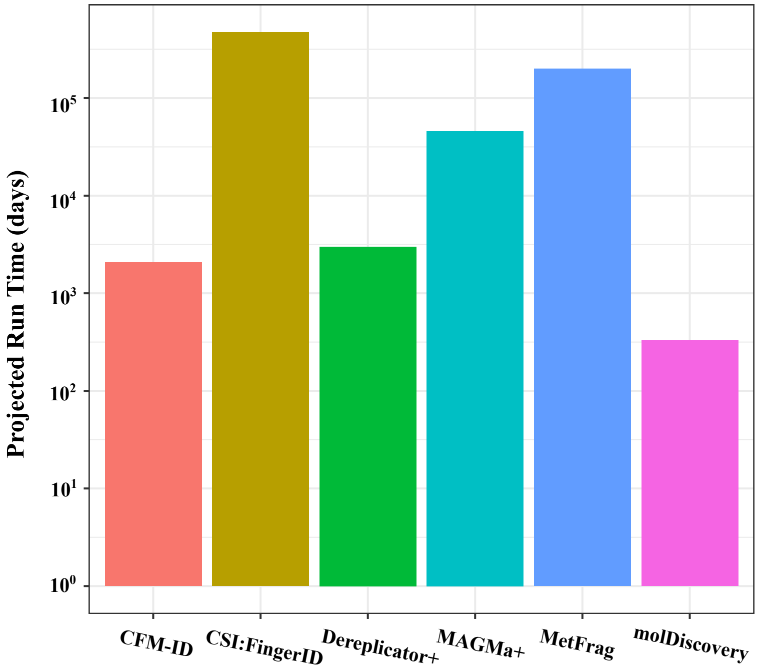

Supplementary Fig 18: Projected running time (preprocessing and search combined) of each method on the combined 46 GNPS spectral datasets listed in Supplementary Table 1 against AllDB. Projection assumes the running time of each method scale with $O(mn)$ where m is the number of compounds in the chemical database and n is the number of spectra. Preprocessing running times are assumed to scale linearly with the number of compounds in the chemical database. It is estimated that it will take molDiscovery 329 days (4 days for preprocessing and 325 days for searching) to finish running, while it will cost CFM-ID 2,068 days (1,402 for preprocessing and 666 day for searching), Dereplicator+ 2997 days (without preprocessing), MAGMA+ 45,988 days, MetFrag 199,139 days, and CSI:FingerID 473,164 days for running.

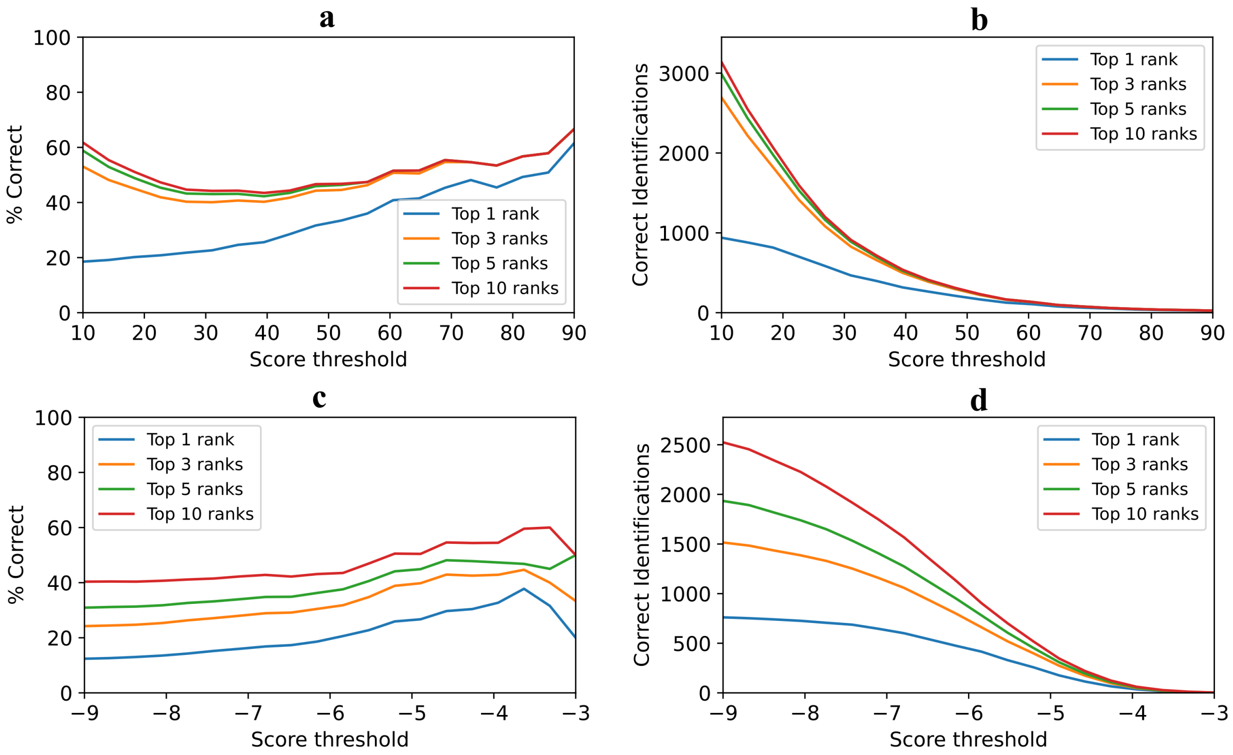

Supplementary Fig 19: **a-b** molDiscovery and **c-d** MAGMa+ accuracy in the search of the MoNA subset (∼6,500 spectra) against bioactive-PubChem (∼1.3M compounds). **a,c** specificity (% correct identifications) and the **b,d** number of correct identifications for top 1, 3, 5 and 10 ranked identifications are shown at different score threshold levels.

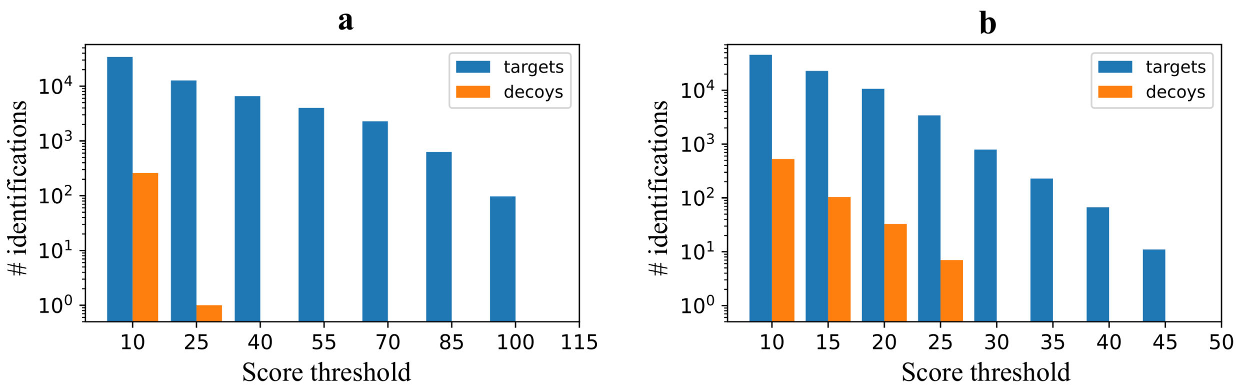

Supplementary Fig 20: Number of identifications in the target and decoy databases at different score thresholds. We show the number of molecule-spectrum matches with **a** molDiscovery and **b** Dereplicator+ in the search of MSV00078604 *Streptomyces* dataset (∼178,000 spectra) against the AntiMarin database (60,908 metabolites). The Y-axis is in log-scale. The number of hits in the decoy database gradually decreases with increasing score. A score threshold corresponding to zero hits in the decoy database separates the most reliable identifications (0% FDR). In this particular case, such score thresholds are 40 and 30 for molDiscovery and Dereplicator+, respectively. Note that these thresholds correspond to almost an order of magnitude more 0% FDR identifications by molDiscovery compared to Dereplicator+.

3 Supplementary Tables.

GNPS ID	Original residue (score)	isobaric residue (score)
CCMSLIB00000854688	ile (108.067)	leu (105.927)
CCMSLIB00000855513	ile (36.7271)	leu (35.7906)
CCMSLIB00000848589	leu (15.3513)	ile (15.7267)

Supplementary Table 1: MolDiscovery scores for three compounds containing leucine or isoleucine and their corresponding isobaric species in the GNPS spectral library.

MassiveID	No. spectra	molDiscovery	spectral library
MSV000078839	403604	0.273	0.002
MSV000078995	818	0.549	0.0
MSV000082831	36566	0.216	0.0
MSV000085214	3284	0.011	0.0
MSV000085180	9095	0.457	0.0
MSV000085023	2372	0.113	0.0
MSV000078836	481548	0.224	0.007
MSV000085003	8526	0.073	0.0
MSV000085123	769	0.0	0.027
MSV000079015	6085	0.508	0.009
MSV000083734	289693	0.886	0.0
MSV000084954	15513	0.819	0.021
MSV000084884	85130	0.287	0.002
MSV000081063	28563	0.751	0.019
MSV000084117	12287	0.392	0.0
MSV000084475	638641	0.491	0.0
MSV000079139	4435	0.003	0.028
MSV000084771	442	0.656	0.0
MSV000085027	426	0.263	0.021
MSV000084945	1550594	0.192	0.015
MSV000081318	6376	0.362	0.0
MSV000085159	188737	0.177	0.0
MSV000080251	1462003	0.433	0.006
MSV000079284	27818	0.041	0.0
MSV000084723	136043	0.065	0.0
MSV000085192	1634	0.234	0.0
MSV000083648	7	0.857	0.0
MSV000081504	607	0.565	0.0
MSV000078891	207413	0.369	0.0
MSV000078847	28615	0.233	0.001
MSV000085158	25606	0.381	0.0
MSV000084674	28854	0.484	0.046
MSV000082285	943	0.525	0.008
MSV000083295	3850	0.227	0.002
MSV000083081	1389	0.672	0.0
MSV000079519	49352	0.315	0.051
MSV000078556	61970	0.123	0.002
MSV000083738	409245	0.531	0.0
MSV000085179	7853	0.012	0.0
MSV000084989	808	0.037	0.0
MSV000080427	12102	0.038	0.0
MSV000082045	1665897	0.405	0.0
MSV000085018	44223	0.933	0.0
MSV000078850	59175	0.317	0.002
MSV000085026	1872	0.014	0.0
MSV000085032	2660	0.072	0.0

Supplementary Table 2: 46 GNPS spectral datasets analyzed in the paper and their annotation rates by molDiscovery and spectral library search. There are 8,013,433 spectra in total. NIST17 spectral library is searched with cosine similarity threshold 0.7, while molDiscovery score threshold is 7 ($\sim 1\% FDR$)

spectral library search		molDiscovery	
compound	#spectral identifications	compound	#spectral identifications
L-Tryptophan	25648	Fenestin_A	67253
Cholic acid	21412	Cyclo(leucylprolyl)_(3R,8aS)-form	41371
Phe-Pro	16856	6-(1-Hydroxy-1-methylethyl)-3-(2-methylp	22781
Trimethoprim	11057	2-Chloro-6-Methyl-Aniline	6354
Compound A	10636	Glycylprolylphenylalanylprolylisoleucine	6156
13-Docosenamide, (Z)-	9157	5-(2-Hydroxy-2-methylpropyl)-3-isopropyl	5991
Ile-Pro-Ile	7978	PE(16:0/17:1(9Z))	5882
DL-Indole-3-lactic acid	7512	cyclo-[Phenylalanyl-prolyl]2	5555
Bis(2-ethylhexyl) phthalate	7239	Nummularine_K_Dihydro	5546
Dibutyl phthalate	6797	cyclo-(proply-proplyl-tyrosyl-tyrosine)	5373

Supplementary Table 3: Most annotated compounds in spectral library search and molDiscovery search in 46 GNPS datasets. The similarity cosine score threshold of spectral library search against NIST17 is 0.7. The FDR threshold of molDiscovery search against AllDB is 1%. Compound A stands for 1,2-Di-(9Z-octadecenoyl)-sn-glycero-3-phosphoethanolamine.

SpecFile	Name	Score	Mass	MZ	RT	Adduct
< 200						
MSV000085018	Chokol A	40.0053	198.162	199.169	1346.53	M+H
MSV000085018	"3,4-Didehydro-beta-ionol"	39.8983	192.151	193.159	878.585	M+H
MSV000085018	4-n-Hexylphenol	39.6969	178.136	179.143	1013.29	M+H
MSV000084674	"Kobifuranone B 2-Deoxo, 5-oxo, 3,4-dihydro"	39.293	196.11	197.117	145.887	M+H
MSV000085158	5-(but-3-en-1-yl)-3-propylfuran-2(5H)-one	38.8054	180.115	181.122	735.623	M+H
MSV000085018	"5(13),7-Megastigmadien-9-one -(E)-form"	38.5856	192.151	193.159	882.017	M+H
MSV000078891	Compound_196.11	38.2996	196.11	197.117	648.557	M+H
MSV000084674	Pestalafuranone G	37.7613	196.11	197.117	150.064	M+H
MSV000085018	"8,10-Pentadecadiene-2,4,6-triyne"	37.4299	196.125	197.132	1321.34	M+H
MSV000084945	"11,12,13-Trinor-2,6-farnesadiene-1,10-diol"	36.6533	198.162	199.17	696.417	M+H
200-400						
MSV000085018	"8Z,11Z,14Z,17Z,20Z,23Z-hexacosahexaenoic acid"	116.156	384.303	385.31	1302.12	M+H
MSV000084475	Raspailol A Raspailol A	110.732	374.282	375.29	353.863	M+H
MSV000084475	3alpha-Hydroxy-5beta-chol-7-en-24-oic Acid	110.701	374.282	375.29	355.213	M+H
MSV000084475	"3-Hydroxychol-11-en-24-oic acid $(3\alpha, 5\beta) - form$"	109.454	374.282	375.29	355.012	M+H
MSV000085018	"25-Dehydrovitamin D3 (5Z,7E)-(3S)-9,10"	107.401	382.324	383.331	1305.55	M+H
MSV000084475	ethyl 10-hydroxy-docosapentaenoate	107.135	374.282	375.29	354.87	M+H
MSV000083738	vanchrobactin	106.099	397.16	398.167	446.19	M+H
MSV000084475	cholacalcioic acid	105.875	372.266	373.274	289.133	M+H
MSV000083738	Ficus Latex peptide 3	105.866	386.264	387.271	1263.63	M+H
MSV000084475	3b-Hydroxy-5-cholenoic acid	105.516	374.282	375.29	353.81	M+H
400-600						
MSV000083738	Antibiotic LL-BM 547alpha	154.272	557.231	558.239	178.786	M+H
MSV000084954	Torularhodin 16'-Alcohol	148.223	550.417	551.423	312.446	M+H
MSV000083738	Heterobactin A	148.005	598.202	599.211	2154.92	M+H
MSV000084954	Alloxanthin Alloxanthin	146.611	564.397	565.401	283.176	M+H
MSV000084954	"(E)-3',4'-didehydro-β,ψ-caroten-16'-ol"	145.624	550.417	551.423	311.062	M+H
MSV000084954	Crocoxanthin	142.812	550.417	551.423	310.387	M+H
MSV000084954	Monadoxanthin 3'-Deoxy	141.02	550.417	551.423	311.472	M+H
MSV000084954	Retrodehydro-g-carotene	139.89	534.423	535.427	313.449	M+H
MSV000083738	13-Hydroxyglucopiericidin A	137.196	593.32	594.327	3300.44	M+H
MSV000083738	"24-Hydroxydammara-20,25-dien-3-one"	135.903	440.365	441.372	2914.57	M+H
600-800						
MSV000084945	Ornibactin C4 N5-Deacyl	212.54	736.397	737.404	176.698	M+H
MSV000081063	Taxlllaid C	195.483	793.531	794.533	612.231	M+H
MSV000083738	Antrimycin D	191.778	727.386	728.394	1949.09	M+H
MSV000084945	a-Substance Ib	184.139	685.391	686.399	247.877	M+H
MSV000081063	Ambactin	183.167	750.406	751.409	395.928	M+H
MSV000081063	Xenobovid B	182.022	793.531	794.535	608.085	M+H
MSV000083738	Monamycin-B3	173.248	677.411	678.417	3688.46	M+H
MSV000083738	Lonicatenamycin	173.163	776.362	777.371	2858.14	M+H
MSV000084884	Alterochromide A	167.987	751.354	752.355	203.68	M+H
MSV000085018	haprolid	167.732	682.431	683.441	2503.76	M+H
800-1000						
MSV000081063	Taxlllaid A	209.695	807.547	808.549	627.514	M+H
MSV000083738	Surugamide G	206.771	883.59	884.594	2615.94	M+H
MSV000079519	E'Champacyclin'	202.923	897.605	898.611	286.793	M+H
MSV000083738	"TG(16:0/20:1(11Z)/18:2(9Z,12Z))"	184.924	882.768	883.776	3829.81	M+H
MSV000078891	Anabaenopeptin NZ857	183.617	857.432	858.435	624.713	M+H
MSV000081063	Xentrivalpeptide A	180.643	859.484	860.487	558.542	M+H
MSV000081063	Szentiamide	171.439	837.406	838.41	509.547	M+H
MSV000079519	Ogipeptin A	168.914	954.597	955.597	269.902	M+H
MSV000081063	Xenobovid C	160.835	821.563	822.564	662.551	M+H
MSV000080251	largamide A methyl ester	158.361	855.438	856.443	200.125	M+H
1000 ≥						
MSV000085018	massetolide J	245.834	1111.67	1112.68	1536.35	M+H
MSV000085018	viscosin	221.532	1125.69	1126.69	1505.12	M+H
MSV000084117	Esperin	206.03	1035.68	1036.69	342.024	M+H
MSV000083738	Actinomycin monolactone	205.893	1272.64	1273.65	3499.08	M+H
MSV000085018	Gacamide A	197.487	1393.84	1394.84	1528.63	M+H
MSV000085192	Sameuramide	192.685	1015.55	1016.55	255.397	M+H
MSV000085018	Antibiotic MA 026 Antibiotic MA 026	189.546	1775.08	888.547	1789.11	M+2H
MSV000085018	Massetolide F	188.566	1125.69	563.852	1612.54	M+2H
MSV000085192	Compound_1001.53	187.785	1001.53	1002.54	260.242	M+H
MSV000083738	callipeltin B	186.724	1030.54	1031.54	3525.33	M+H

Supplementary Table 4: Top identifications of molDiscovery in the 46 datasets in different mass ranges. Compound_196.11 represents 3-(1-Hydroxyhexyl)-5-methylene-2(5H)-furanone. Compound_1001.53 represents "3-acetamido-22-benzyl-10-<1<(3-hydroxy-4-methyl-2-propionamidopentanoyl)oxy¿-2-methylpropyl>-4-isopropyl-7-(1-methoxyethyl)-19-methylene-8,13,14,16,20-pentamethyl-1,5-dioxa-8,11,14,17,20-pentaazacyclodocosane-2,6,9,12,15,18,21-heptone"

Method	Projected Running time (d-h:m:s) on MoNA
molDiscovery	Preprocessing - 7-3:08:04 Running - 11:25:40
CFM-ID	Preprocessing - 2522-21:42:37 Running - 23:26:15
Dereplicator+	4-9:25:53
MAGMa+	67-9:41:16
MetFrag	291-21:01:36
CSI:FingerID	693-12:19:03

Supplementary Table 5: Projected running times of all methods on searching the MoNA subset (∼6,500 spectra) against bioactive-PubChem (∼1.3M compounds). Projections assume that all the methods scale with $O(mn)$ for search and with $O(m)$ for preprocessing where m is the number of compounds in the chemical database and n is the number of spectra being searched. Projections are computed based on the running times recorded when searching GNPS against DNP.

location	product	gene
58..732	TetR family transcriptional regulator	
complement(906..1148)	hypothetical protein	
complement(1767..2738)	sugar ABC transporter permease	
complement(2735..3817)	sugar ABC transporter permease	
complement(3814..5337)	sugar ABC transporter ATP-binding protein	
complement(5416..6399)	LacI family transcriptional regulator	
complement(7105..8094)	LacI family transcriptional regulator	
complement(8548..10116)	alpha-N-arabinofuranosidase	abfA_1
complement(10249..11811)	alpha-N-arabinofuranosidase	abfA_2
12286..14997	hypothetical protein	
complement(15176..16537)	hypothetical protein	
complement(17549..17746)	hypothetical protein	
complement(17746..18498)	hypothetical protein	
complement(18529..19776)	cytochrome P-450 like protein	
complement(19782..19997)	MbtH protein	mbtH
complement(20001..28664)	hypothetical protein	
28826..29341	hypothetical protein	
complement(30212..43642)	hypothetical protein	
complement(43747..46140)	hypothetical protein	
complement(46246..46617)	hypothetical protein	
complement(46920..48608)	hypothetical protein	
48820..50400	peptide ABC transporter substrate-binding protein	
50397..51383	peptide ABC transporter permease	
51386..53278	peptide ABC transporter ATP-binding protein	
53275..54078	hypothetical protein	
54075..55379	oxidoreductase	
complement(55369..56451)	hypothetical protein	
complement(56636..58819)	hypothetical protein	
59088..60437	glutamine synthetase	
60602..60793	hypothetical protein	
complement(61351..61728)	hypothetical protein	
complement(61980..62429)	cyclase	
complement(62736..63191)	hypothetical protein	

Supplementary Table 6: Gene annotation of dinghupeptin family BGC. The genes are annotated by DFAST.

location	product	gene
1..297	hypothetical protein	
501..1964	6-aminohexanoate-cyclic-dimer hydrolase	
complement(2177..3001)	DDE transposase	
complement(3138..3452)	hypothetical protein	
3583..3984	hypothetical protein	
4543..4998	hypothetical protein	
5019..5648	hypothetical protein	
complement(5972..6196)	hypothetical protein	
6286..6558	hypothetical protein	
6885..7265	hypothetical protein	
complement(8267..8671)	hypothetical protein	
complement(8709..9554)	hypothetical protein	
10227..11204	oxidoreductase	
complement(12255..13619)	MFS transporter	
complement(13629..15032)	hypothetical protein	
15240..16388	hypothetical protein	
16585..17538	"2,3-diaminopropionate biosynthesis protein SbnA"	cysM
17553..18572	"2,3-diaminopropionate biosynthesis protein SbnB"	ocd
18576..18785	hypothetical protein	
18782..19519	thioesterase	
19534..24468	hypothetical protein	
24564..27824	hypothetical protein	
27821..28621	hypothetical protein	
28746..29927	hypothetical protein	
complement(30021..31859)	SARP family transcriptional regulator	
complement(32071..33096)	2-oxobutyrate oxidase	
complement(33093..33782)	7-cyano-7-deazaguanine synthase	queC
complement(33784..34764)	hypothetical protein	
complement(34847..35437)	GTP cyclohydrolase 1	folE
complement(35475..35753)	"6-carboxy-5,6,7,8-tetrahydropterin synthase"	
complement(35875..36588)	7-carboxy-7-deazaguanine synthase	queE
complement(36602..37384)	hypothetical protein	
complement(37758..40760)	SARP family transcriptional regulator	
complement(40780..40947)	hypothetical protein	
41800..42135	hypothetical protein	
complement(42775..43035)	hypothetical protein	
complement(43145..43909)	thiazole biosynthesis protein ThiJ	
43956..44909	AraC family transcriptional regulator	
45336..45962	resolvase	
46065..46439	hypothetical protein	
47147..47404	hypothetical protein	
47401..48699	MFS transporter	
48973..49656	hypothetical protein	
50297..50692	hypothetical protein	
51382..51702	hypothetical protein	
complement(52074..53123)	L-asparagine oxygenase	
complement(53230..53448)	protein mbtH	
complement(53519..60676)	hypothetical protein	
complement(60673..67518)	hypothetical protein	
complement(67577..83815)	hypothetical protein	
complement(84104..84379)	hypothetical protein	
complement(84487..84762)	hypothetical protein	
84866..85963	hypothetical protein	
85973..86224	hypothetical protein	
86352..87116	hypothetical protein	
complement(87317..87841)	integrase	

Supplementary Table 7: Gene annotation of lipopeptin family BGC. The genes are annotated by DFAST.

location	product	gene
3271..4461	amidase	amiB1
complement(4525..5523)	ABC transporter substrate-binding protein	
5693..6487	cobalamin/Fe3+-siderophore ABC transporter ATP-binding protein	
complement(6564..7784)	hypothetical protein	
8076..8843	hypothetical protein	
8871..9560	methyltransferase	
9541..9819	hypothetical protein	
complement(9836..10813)	hypothetical protein	
complement(10948..12174)	hypothetical protein	
12425..12667	hypothetical protein	
12805..13461	hypothetical protein	
complement(13535..14590)	hypothetical protein	
15272..16558	MFS transporter	
16776..17003	hypothetical protein	
17478..18545	prenyltransferase	
18542..19795	aminotransferase	
complement(19821..21287)	cytochrome P450	
complement(21284..21907)	ATP-binding protein	
complement(21888..22259)	hypothetical protein	
complement(22256..22678)	dynein regulation protein LC7	
complement(22697..24088)	ATPase	
complement(24655..25614)	hypothetical protein	
complement(25702..25896)	hypothetical protein	
complement(26542..26922)	hypothetical protein	
27175..28071	transcriptional regulator	
28263..28982	ABC transporter ATP-binding protein	
complement(29064..29714)	hypothetical protein	
30045..30314	hypothetical protein	
complement(30305..30679)	hypothetical protein	
31032..32432	3-isopropylmalate dehydratase large subunit	leuC
32447..33073	3-isopropylmalate dehydratase small subunit	leuD
33073..34284	aminotransferase	
34281..35309	protein AmbC	ambC
35306..36574	MFS transporter	
complement(36626..37270)	DNA-binding response regulator	
complement(37258..38514)	histidine kinase	
38699..39613	ABC transporter	
39618..42188	ABC transporter permease	
42900..44093	serine hydrolase	
44203..50637	hypothetical protein	
50641..63150	hypothetical protein	
63188..67786	hypothetical protein	
67842..68594	thioesterase	
68962..70464	tryptophan halogenase	
70502..71761	hypothetical protein	
71758..72279	FMN reductase	
72332..73159	amidinotransferase	
73203..74879	2-isopropylmalate synthase	leuA
74962..75777	indole-3-glycerol phosphate synthase 1	trpC1
complement(75749..76807)	anthranilate phosphoribosyltransferase 1	trpD1
77032..78459	GntR family transcriptional regulator	
complement(78468..79040)	glutamine amidotransferase	
complement(79037..80647)	hypothetical protein	
80857..82200	phospho-2-dehydro-3-deoxyheptonate aldolase	aroH
82399..83418	3-oxoacyl-ACP synthase	fabH
83448..85319	hypothetical protein	
85433..90103	hypothetical protein	
90100..98496	hypothetical protein	

Supplementary Table 8: Gene annotation of lonicatenamycin family BGC. The genes are annotated by DFAST.

bond type	all	marine	terrestial
C-H	2050594(45.4%)	196820(45.6%)	1853774(45.4%)
C-C	1283757(28.4%)	121283(28.1%)	1162474(28.4%)
C-O	356218(7.8%)	34168(7.9%)	322050(7.8%)
C=C	222100(4.9%)	23698(5.4%)	198402(4.9%)
C-N	183013(4.0%)	16505(3.8%)	166508(4.0%)
C=O	140625(3.1%)	11868(2.7%)	128757(3.1%)
O-H	138568(3.0%)	14552(3.3%)	124016(3.0%)
N-H	82728(1.8%)	6130(1.4%)	76598(1.8%)
C=N	11747(0.2%)	1109(0.2%)	10638(0.2%)
C-S	9179(0.2%)	998(0.2%)	8181(0.2%)
C-Br	7998(0.1%)	1403(0.3%)	6595(0.1%)
C-Cl	6191(0.1%)	824(0.1%)	5367(0.1%)
S=O	5007(0.1%)	447(0.1%)	4560(0.1%)

Supplementary Table 9: Bond type frequencies in AntiMarin database. All, marine and terrestial stands for all, marine and terrestial compounds in AntiMarin database respectively. Among top 9 most frequent bonds in natural products, only C-C, C-O and C-N do not have hydrogen or double bond. Moreover, as the frequencies of C-S, C-Br and C-Cl are less than 0.2%, and there is few training data in GNPS spectral library for these bond types, we only focus on C-C, C-O and C-N in molDiscovery. These statistics is from Supplementary Table 4 of Dereplicator+.

Fragment A	Fragment B	Hydrogen rearrangement
C	C	A+H B-H or A-H B+H
C	N	A-H B+H
C	O	A-H B+H
C	S	A-H B+H
C	P	A+H B-H
O	P	A+H B-H

Supplementary Table 10: Rearrangement rules used in molDiscovery. MolDiscovery associates the hydrogen rearrangement rules with the type of bond that is fragmented. All the bonds in the table are single bonds. For example, whenever C-O bond is disconnected, a -H mass shift is considered for the fragment on the carbon side, and +H mass shift is considered for the fragment on the oxygen side. When a C-C bond is disconnected, both rearrangement scenarios are considered.
